# Epigallocatechin-3-gallate preferentially induces aggregation of amyloidogenic immunoglobulin light chains

**DOI:** 10.1038/srep41515

**Published:** 2017-01-27

**Authors:** Manuel Hora, Martin Carballo-Pacheco, Benedikt Weber, Vanessa K. Morris, Antje Wittkopf, Johannes Buchner, Birgit Strodel, Bernd Reif

**Affiliations:** 1 Center for Integrated Protein Science at Department Chemie, Technische Universität München Lichtenbergstrasse 4, 85747 Garching, Germany; 2 Helmholtz-Zentrum München (HMGU), Ingolstädter Landstr. 1, 85764 Neuherberg, Germany; 3 Institute of Complex Systems: Structural Biochemistry at Forschungszentrum Jülich, 52425 Jülich, Germany

## Abstract

Antibody light chain amyloidosis is a rare disease caused by fibril formation of secreted immunoglobulin light chains (LCs). The huge variety of antibody sequences puts a serious challenge to drug discovery. The green tea polyphenol epigallocatechin-3-gallate (EGCG) is known to interfere with fibril formation in general. Here we present solution- and solid-state NMR studies as well as MD simulations to characterise the interaction of EGCG with LC variable domains. We identified two distinct EGCG binding sites, both of which include a proline as an important recognition element. The binding sites were confirmed by site-directed mutagenesis and solid-state NMR analysis. The EGCG-induced protein complexes are unstructured. We propose a general mechanistic model for EGCG binding to a conserved site in LCs. We find that EGCG reacts selectively with amyloidogenic mutants. This makes this compound a promising lead structure, that can handle the immense sequence variability of antibody LCs.

Systemic antibody light chain amyloidosis (AL amyloidosis) is a systemic disease caused by amyloid fibril formation of free immunoglobulin light chains (LCs) in the serum^1^. Patients typically suffer from a non-symptomatic monoclonal plasma-cell dyscrasia, resulting in an overproduction and secretion of clonal antibody LCs. These LCs, also referred to as Bence-Jones-proteins^2^, may deposit as fibrils in all inner organs, mainly heart, kidneys and nerves^3^,^4^. Most patients die from cardiac amyloid deposits^5^ and despite improvements, mortality remains high, with 4 year overall-survival of 42%^6^.

Contemporary therapy is targeted against the underlying B-cell dyscrasia, e.g. by application of high-dose melphalan^7^ and autologous cell transplantation^8^. Alternatively, bortezomib is a promising therapeutic agent^9^,^10^. Although these treatments are effective, they are poorly tolerated and cause severe side-effects^11^. In addition, these therapies do not affect already secreted serum free LCs or amyloid deposits.

Targeting the amyloidogenic LCs is a difficult task, due to the tremendous variety of antibody sequences. It is still unclear, which properties render some sequences prone to amyloid formation. In most cases, only the variable domain of the LCs is found in the fibrils, but sometimes also the constant domain is present^12^,^13^. The linker between both domains also affects fibril formation^14^. Certain germline genes are frequently associated with AL amyloidosis and may also affect organ involvement^15^,^16^. Thermodynamic stability was shown to be an important factor for fibril formation. Unstable sequences have higher propensity to aggregate, but too low stability can also prevent fibrillogenesis^17^, indicating that partially unfolded states are involved in the pathway to fibril formation. These intermediates might be populated *in vitro* at acidic or otherwise destabilising conditions^18^,^19^,^20^,^21^. In addition, a plethora of mutations were shown to be associated with fibril formation^22^,^23^,^24^,^25^,^26^,^27^.

Due to the variety of different AL protein sequences, the search for therapeutic agents interfering with LC aggregation is a daunting task. Recently, a promising study presented CPHPC as an agent targeting serum amyloid P, a non-fibrillar component of all systemic amyloidosis fibril deposits^28^. In addition, methylene blue and sulfasalazine have been suggested to prevent fibril formation of immunoglobulin LCs by stabilising the more stable LC dimer^29^.

Epigallocatechin-3-gallate (EGCG) has already been shown to interact with other amyloid proteins like α-synuclein^30^, amyloid-β^31^,^32^, huntingtin^33^, IAPP^34^,^35^, transthyretin^36^,^37^ tau^38^ and SEVI^39^. For these proteins, detailed mechanistic insights are available, highlighting the role of oxidation of EGCG^40^, redirection to non-toxic species^41^ or remodeling of fibrils^42^. EGCG has already been studied in the context of AL amyloidosis. It redirects the LCs into partially SDS-stable aggregates^43^ and also causes a considerable delay of aggregation kinetics by interactions with the native state^44^. These *in vitro* studies support preliminary *in vivo* reports on the efficacy of EGCG against AL amyloidosis in *C. elegans*^45^ and in human patients^46^,^47^,^48^. Two clinical trials in phase II are ongoing (EpiCardiAL, ClinicalTrials.gov Identifier: NCT01511263; TAME-AL, ClinicalTrials.gov Identifier: NCT02015312).

In this study, we report on the mechanism of EGCG interacting with the LC V_L_ domain of MAK33. The murine MAK33 antibody targets muscle-type creatinine kinase and its V_L_ domain is used here as a model system to investigate amyloid aggregation. The κ-type MAK33 V_L_ domain has been studied in detail regarding structure^49^ and biophysical properties^19^,^20^. Several point mutants have been shown to form amyloid fibrils. The strongly destabilising I2E mutant can form fibrils at native conditions^25^. Both the S20N and the D70N substitutions are sufficient to enable fibril formation under destabilising conditions, e.g. sonication or addition of SDS^26^. The latter two mutants are especially interesting, since they display the same thermodynamic stability as the WT protein.

Binding of EGCG was investigated using solution-state nuclear magnetic resonance (NMR) spectroscopy, site-directed mutagenesis and docking studies. Comparison of aggregation kinetics with different mutants indicates increased affinity to LCs with higher amyloidogenicity. Solid-state NMR as well as infrared spectroscopy show that EGCG-V_L_ coprecipitates are mostly unstructured, with only the binding site being structurally well defined.

## Results

### EGCG binds specifically to proline 59

**Figure 1:**
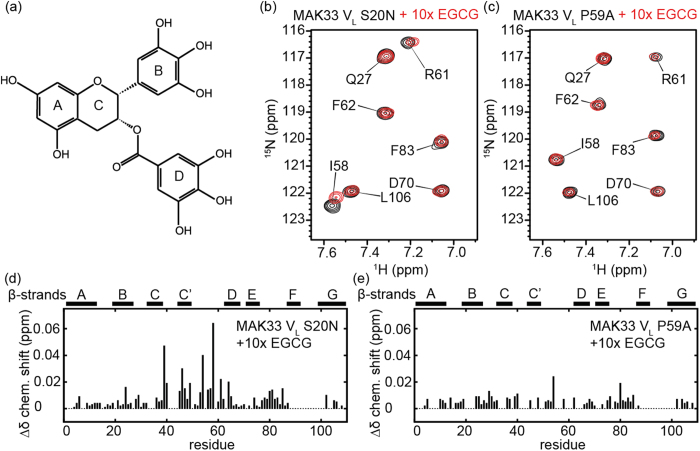
(**a**) Epigallocatechin-3-gallate (EGCG). (**b**,**c**) ^1^H,^15^N NMR correlation spectra of MAK33 V_L_ S20N and V_L_ P59A in presence and absence of EGCG. For S20N, addition of EGCG caused chemical shift perturbations for I58 and R61. Mutation of P59 prevented these shift changes. (**d**,**e**) Chemical shift perturbations for the S20N and P59A mutants in the presence of EGCG. Gaps indicate residues, which are not assigned.

To derive a general binding principle for EGCG to V_L_ domains, we characterised its interaction with different MAK33 V_L_ protein sequences. We used the S20N mutant for initial experiments to study the interaction with EGCG (Fig. 1a), because its fibril formation can be easily controlled by experimental settings. Addition of EGCG to the S20N variant resulted in moderate, but reproducible chemical shift perturbations (Fig. 1b,d; see SI 1, 2 for further details), with strongest effects on K39, L46, S54, I58 and R61. These residues are located close to each other in the structure. While EGCG is expected to cause strong chemical shift changes due to its aromatic ring systems, the small chemical shift perturbations observed here are in agreement with previous literature^50^. In addition to chemical shift changes, titration of EGCG also caused visible precipitation.

EGCG is known to bind strongly to proline-rich peptides^51^. Since proline lacks the amide hydrogen atom, ^1^H,^15^N-HSQC NMR experiments fail to detect this residue. However, mapping of chemical shift perturbations on the structure shows most of the affected residues being close to P59. Therefore, P59 is a likely candidate for interaction with EGCG in the V_L_ domain (Fig. 1b,d). To validate this interaction, we mutated this proline to alanine. P59 is located in a loop area. Thus, the mutation is unlikely to change the structure of the domain. Comparison of NMR chemical shifts revealed only minor differences between the WT, the amyloidogenic S20N and the P59A mutant, confirming this assumption (data not shown). The mutation was, however, sufficient to suppress most chemical shift perturbations induced by EGCG (Fig. 1c,e), confirming the important role of P59 for EGCG binding. At the same time, we find that the P59A mutant is still precipitated by EGCG (SI 3).

### EGCG can bind at two distinct sites

**Figure 2:**
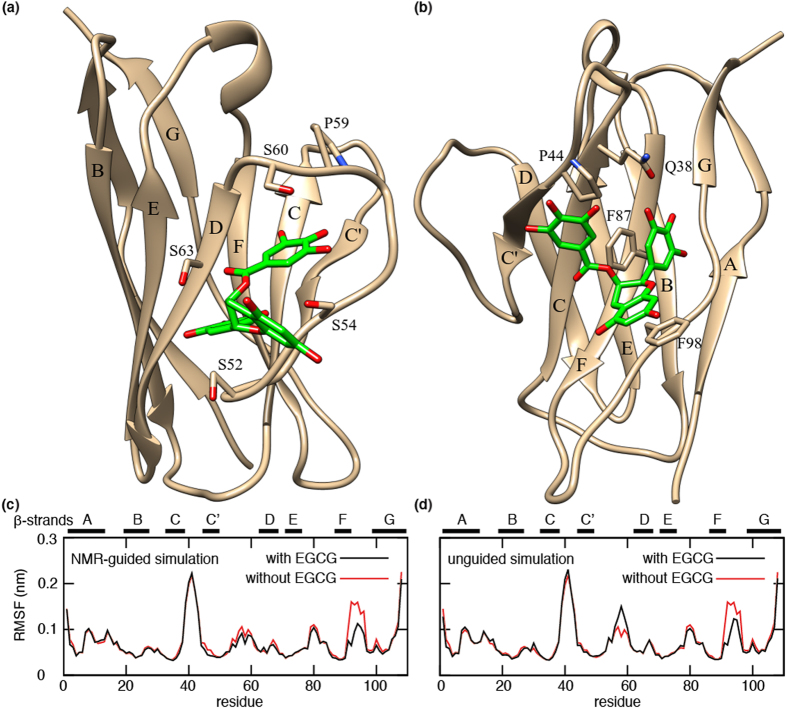
Most stable complex structure from (**a**) NMR-guided and (**b**) unguided simulations of MAK33 V_L_ with EGCG. Root mean square fluctuations (RMSF) for each residue according to MD simulations starting from complexes obtained from (**c**) NMR-guided and (**d**) unguided docking simulations.

To further characterise the binding site, we performed docking studies, both with and without guidance from NMR chemical shift perturbations (Fig. 2). Based on the NMR experiments, we first ran docking experiments with predefined contacts to I58 and P59. This NMR-guided docking suggested a binding site for EGCG involving residues S52, S54, S60, S63 and G64 (Fig. 2a). However, the subsequent MD simulation showed that EGCG is not tightly bound at that position. It fluctuated around the starting structure and eventually dissociated within the 500 ns simulation. This indicates low affinity binding.

In a complementary simulation, we docked EGCG to MAK33 V_L_ without experimental restraints. In this case, EGCG bound to another site, with strong interactions with Q38, P44, F87 and F98 (Fig. 2b). During the subsequent 500 ns MD trajectory, EGCG was more stably bound at that site, indicating a higher binding affinity compared to binding around residues I58 and P59. The stable binding in this position originates from π-π stacking interactions between ring A of EGCG and F98, ring B and F87, a hydrogen bond between one of the hydroxyl groups of ring B and Q38, which is stable during the MD simulation, and electrostatic interactions between R45 and one of the hydroxyl groups of ring D of EGCG. In the binding mode obtained from NMR-guided docking, the interactions between EGCG and MAK33 V_L_ are generally less pronounced and especially the π-π stacking interactions are missing. Moreover, the stable binding predicted by unguided docking occurs in a similar position as reported for 6aJL2 R24G^44^, suggesting a common binding site for different immunoglobulin V_L_ domains. In both the NMR-guided and unguided simulations, we observed a decrease in protein flexibility in the region around residues 92–94 as a result of EGCG binding (Fig. 2c,d). In the unguided simulation, there is also an increase of flexibility around residue P59, which is not observed in the guided simulation. This is an interesting observation, as in the unguided simulation EGCG does not bind directly to P59, yet its dynamics is affected by EGCG binding in a neighbouring region on the V_L_ surface. This change in P59 dynamics may give rise to the chemical shift perturbations in that area as observed for MAK33 V_L_ S20N in the presence of EGCG (Fig. 1d).

### Interaction with proline 44 leads to precipitation

Using solution-state NMR, we were not able to probe the EGCG binding experimentally, since the residues located in the predicted binding site are only poorly visible or cannot be detected at all. This is caused by dimerisation-related dynamics. Homodimerisation is frequently observed for V_L_ domains^52^. This typically weak interaction results in exchange broadening of the NMR resonances for residues involved in the dimer interface^21^. These exchange dynamics can also be observed for MAK33 V_L_. The EGCG binding site observed by unguided docking is located in this dimer interface.

**Figure 3:**
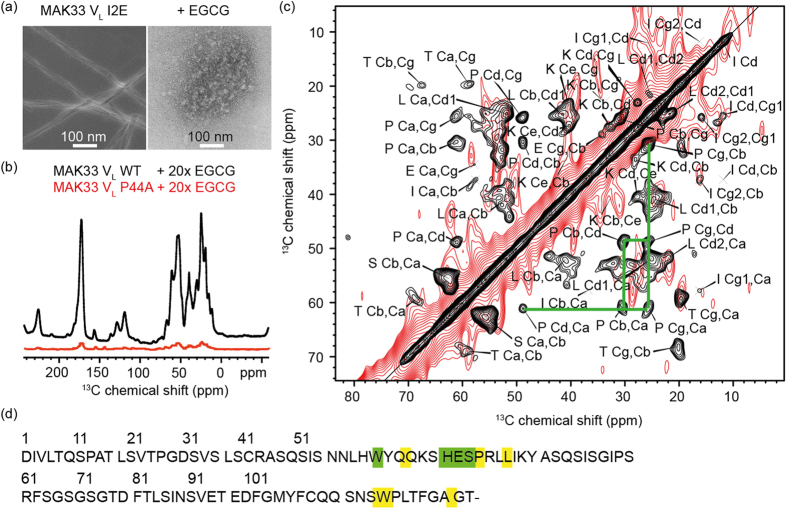
(**a**) Precipitates of MAK33 V_L_ I2E visualised by TEM. To enable use of native-like conditions for fibril formation, the highly amyloidogenic I2E mutant was employed. Fibrils were grown by incubating a 50 μM protein solution at a temperature of 37 °C and agitating the sample at 120 rpm (left). The sample with EGCG-induced precipitates (right) was treated identically, but in the presence of a 10-fold excess of EGCG. (**b**) MAS solid-state NMR analysis of coprecipitates of EGCG with the WT or the P44A mutant. The P44A precipitates yield a drastically reduced signal intensity in ^1^H,^13^C-cross polarization experiments. (**c**) 2D ^13^C,^13^C PDSD correlation spectrum of coprecipitates of MAK33 V_L_ WT and EGCG (black). Nine spin systems could be identified, which presumably originate from residues close to the EGCG binding site. In the lower right part of the symmetric spectrum, the proline spin system is marked with green lines. The NMR spectrum of the P44A variant prepared in the same way (red) does not yield a comparable cross peak pattern. (**d**) Assigned spin systems of EGCG-induced WT-V_L_ aggregates. The identified spin systems were assigned to those residues, which are closest to the EGCG binding site in the unguided docking structure (Fig. 2b; details in SI 4). Unambiguous assignments are shown in green, ambiguous assignments in yellow.

In order to confirm this binding site experimentally, we collected the EGCG-protein coprecipitate (Fig. 3a) and conducted solid-state NMR experiments. We performed a ^13^C,^13^C correlation experiment, which relies on dipolar couplings. Since anisotropic interactions are averaged to zero in dynamic regions, this experiment displays only the rigid parts of the sample. The spectral quality reflects the homogeneity and rigidity of the sample. We determined a ^13^C linewidth on the order of 250 Hz to 300 Hz, which indicates a high degree of structural heterogeneity in the precipitate. By contrast, structured amyloid aggregates display ^13^C linewidths of approximately 100 Hz^53^. Still, we obtained a spectrum, in which we could assign the spin systems of nine amino acids (Fig. 3c). The observed amino acids include arginine (only side chain), glutamate, isoleucine, leucine, lysine, proline, serine, threonine and tyrosine (only side chain). Since only nine out of 108 amino acids were visible, we assume that the protein is unfolded in the precipitate, which is in agreement with the broad resonance lines mentioned above. The visible resonances originate from residues, which are presumably rigidified due to interactions with EGCG. Their assignments provide important clues to identify the EGCG binding site in the precipitates.

Due to resonance broadening, sequential assignment was not feasible for EGCG-induced MAK33 V_L_ WT aggregates. However, the observed spin systems allow already to restrict the potential binding site by making use of the primary sequence of the protein and the docking experiments. The proline Cβ and Cγ resonances have a chemical shift difference of 5 ppm, which is a clear indication for a trans conformation^54^. Out of the five prolines in MAK33 V_L_, two prolines are in cis conformation in the native state (P8 and P95)^19^,^49^. We can assume that binding of EGCG does not alter the proline conformational state, as the trans conformation is fixed by the interaction with EGCG. This leaves three prolines as potential binding sites: P15, P44 and P59. Mapping of the identified amino acids on the sequence and the unguided docking structure suggest P44 as the preferred binding site (Fig. 3d). Except for threonine, all identified amino acids are close to P44 in the structure (SI 4). Isoleucine and threonine have very weak intensities in the spectrum, suggesting that they are further away from the binding site (which is the case for I48), or located in other flexible regions.

As a control, the solid-state NMR experiment was repeated with the coprecipitate of EGCG and the P44A mutant. Even though sample preparation was identical, the signal intensity in ^13^C cross polarization 1D experiments was lower compared to the experiment carried out with the WT protein (Fig. 3b). The difference in intensities, corrected for slightly different amounts of sample in the initial setup for precipitation, corresponds to a factor of ca. 15, indicating that the P44A mutant is precipitated with considerably lower efficiency. The ^13^C,^13^C correlation experiment that was acquired for these precipitates hardly shows any cross peaks even after three days of measurement time (Fig. 3c). These observations, together with the docking experiments, suggest a crucial role of P44 for binding EGCG.

### EGCG interacts stronger with amyloidogenic mutants

To gain insights into the mode of action of EGCG, we quantified the kinetics of EGCG-induced precipitation using solution-state NMR. First, we monitored precipitation at different concentrations of EGCG. For this experiment, we used the S20N mutant, which has the same thermodynamic stability as the WT, but displays increased fibrillogenicity^26^. In the control experiment, we added water to the sample. In this case, we saw only a fast initial decrease of protein signal intensity induced by mixing of the sample and subsequent unfolding of a fraction of the protein. This initial decrease was similar in all further experiments.

**Figure 4:**
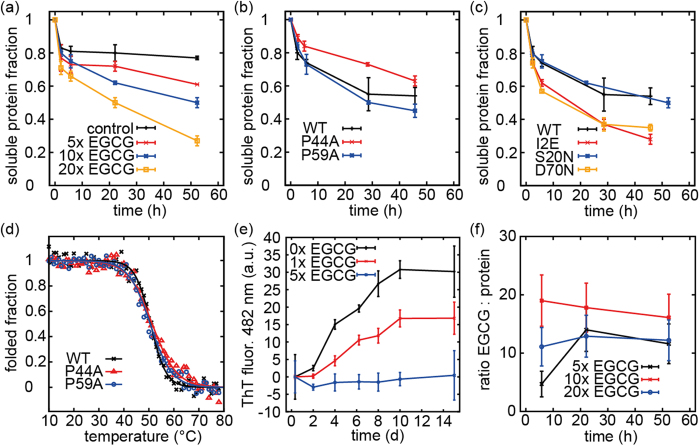
(**a**) Aggregation kinetics of MAK33 V_L_ S20N induced by EGCG. ^1^H NMR was employed for quantification. (**b**) Precipitation of the V_L_ variants with altered EGCG binding properties in presence of a 10-fold molar excess of EGCG. (**c**) The more amyloidogenic mutants D70N and I2E precipitate faster in presence of a 10-fold excess of EGCG, although D70N has the same thermodynamic stability as the WT. (**d**) Thermal unfolding of MAK33 V_L_ P44A and P59A. The mutations do not affect melting temperatures, as confirmed by CD thermal transitions. (**e**) ThT fibril formation assays for V_L_ I2E with and without EGCG. (**f**) EGCG to protein V_L_ S20N molar ratio in the aggregates quantified by ^1^H NMR signal intensities.

Over the course of more than two days, we saw hardly any decrease in intensity in the control experiment, whereas increasing amounts of EGCG resulted in higher degrees of precipitation (see Fig. 4a). A 5-fold molar excess of EGCG caused reduction of the protein signal by 26% over two days, while 20-fold excess reduced the signal by 65%, indicating a concentration dependent mode of action of EGCG.

Next, we investigated the influence of the two prolines P44 and P59 on the interaction with EGCG (Fig. 4b). As expected from our previous experiments, the P44A mutant precipitated slower than the WT in the presence of EGCG, although the effect was not as pronounced as in the solid-state NMR experiments reported above. The P59A mutant precipitated slightly faster than the WT. This might be a consequence of different dynamics due to the mutation. Neither of these mutations had strong effects on protein stability, as determined by temperature-induced unfolding transitions monitored by CD spectroscopy. The *T*_*m*_ values for the WT protein, P44A and P59A were determined to be 50.7 °C, 51.3 °C and 50.1 °C, respectively (Fig. 4d). Of note, the thermal transitions are irreversible. Thus, the T_m_ values are apparent values. The slight increase of *T*_*m*_ for P44A cannot account for the drastic differences observed regarding the precipitation behaviour in presence of EGCG.

In the following, we compared V_L_ mutants with different propensities to form amyloid fibrils^25^,^26^ with respect to their interactions with EGCG. The S20N and the D70N mutants both have the same melting temperature and thermodynamic stability as WT V_L_. However, while WT V_L_ forms fibrils only at pH 2, these mutants form amyloid aggregates at native pH, upon ultrasonication^26^. The I2E mutant has a drastically reduced thermodynamic stability and forms fibrils at native-like conditions without addition of destabilising chemicals^25^. We conducted precipitation experiments with all variants, adding a 10-fold excess of EGCG (Fig. 4c). The S20N mutant displayed the same precipitation kinetics as the WT, while the D70N and I2E proteins precipitated considerably faster. Solid-state NMR experiments revealed that these precipitates are mostly unstructured. As I2E is far less stable, it might be expected that this variant forms unstructured aggregates with EGCG faster than the WT. The D70N mutant, in contrast, has the same stability as the WT, but is more likely to undergo a transition to fibrils. This indicates, that EGCG not just precipitates all antibody LCs, but has a higher affinity for the more amyloidogenic species. The S20N mutant’s slow precipitation might be related to different pathways for amyloid fibril formation. Since the S20N mutant differs both from the WT and the D70N mutant with respect to the m-value for GdmCl denaturation^26^, it might expose different sites during fibril formation and thus react only moderately with EGCG.

To probe amyloid fibril formation and ThT binding^55^, we employed the most amyloidogenic variant. In the absence of EGCG, the I2E variant started immediatelly to form fibrils and reached the plateau phase after 10 days (Fig. 4e). At a 1:1 molar ratio, EGCG already considerably reduced the amount of fibrils. A 5-fold excess of EGCG was enough to quantitatively prevent fibril formation. We are aware of the fact that EGCG changes the fluorescence spectrum of ThT. Therefore, the results were analysed with caution. In agreement with the ThT results, we found no fibrils in TEM experiments (Fig. 3a). In addition, Fourier-transform infrared (FTIR) spectroscopy confirmed the non-fibrillar structure of the EGCG-protein coprecipitates (SI 5). Upon addition of EGCG, the kinetics of fibril formation remained unperturbed, whereas the amount of formed fibrils is significantly reduced. A comparison between the ThT kinetics and the precipitation kinetics (Fig. 4c) suggests, that EGCG-induced aggregates form much faster than amyloid fibrils.

Finally we quantified stochiometries of the precipitate-EGCG complexes. Pelaez-Aguilar *et al*. have reported a 1:1 stochiometry for binding of EGCG to 6aJL2 R24G in solution, determined by isothermal titration calorimetry^44^. However, the stochiometry of the coaggregates was not quantified yet. We compared the reduction of the ^1^H NMR signal intensities of both MAK33 V_L_ S20N and EGCG over time (Fig. 4f). The stochiometries vary both with time and with respect to the initial stochiometry in solution. We find that 10 to 20 molecules of EGCG co-aggregate per precipitated protein molecule. While the initial binding in solution might be 1:1, more EGCG is found in the coaggregates. As the aggregates were shown to be unstructured, more EGCG seems to be recruited during later stages of precipitation. This might indicate, that precipitation is caused be interactions between EGCG molecules.

## Discussion

We have shown how the polyphenol EGCG interacts with the murine κ-type MAK33 V_L_ antibody domain. EGCG was already shown to bind to 6aJL2 R24G, which is a highly amyloidogenic λ-type V_L_ sequence^44^. Andrich *et al*. reported anti-amyloidogenic activity of EGCG for another LC sequence, 1AL1^43^. This suggests, that EGCG interacts with both κ- and λ-type AL sequences.

**Figure 5:**
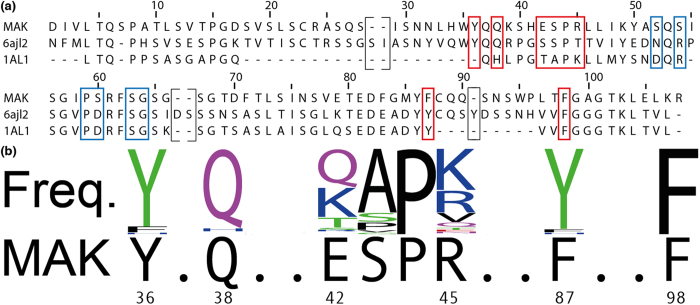
(**a**) Sequence alignments for MAK33^49^, 6aJL2^44^ and 1AL1^43^. EGCG was shown to interact with all of these sequences. Residues in the vicinity of the P44 binding site are marked with red boxes. Residues involving the P59 binding site are marked in blue. Brackets exclude gaps in MAK33 from the residue numbering. (**b**) Frequency plot of residues in the binding site for human V_L_ sequences. Most of the residues of the P44 binding site are conserved in human V_L_ sequences. The frequency plot was created using amino acid frequencies from more than 11,000 human LC sequences (taken from abYsis 2.3.3; http://www.bioinf.org.uk/abysis/).

Many residues important for the interaction with EGCG are conserved between MAK33, 6aJL2 and 1AL1 (Fig. 5a). The highest conservation is obtained for P44 and the aromatic residues Y36, F87 (corresponding to tyrosine in 6aJL2 or 1AL1) and F98 (residue numbering as in MAK33). This is valid even if the comparison is expanded to a very large set of human V_L_ domains (Fig. 5b). In addition to the highly conserved residues Y36, P44, F87 and F98, a basic residue is found at position 45 (arginine in case of MAK33). Positions 42 and 43 are more variable. Considering that also 6aJL2 and 1AL1 differ from MAK33 in these positions, they seem to be less crucial for EGCG binding. Despite minor differences in the primary sequences, the EGCG binding site reported for 6aJL2 matches well with the P44 site described here^44^. Even though we used EGCG in excess, the match between binding sites for MAK33 and 6aJL2 indicates specific binding. For 6aJL2, an affinity of K_A_ = 1.35 × 10^4^ M^−1^ has been reported^44^. Considering the conservation of the binding site, the vast majority of V_L_ domains should be able to bind EGCG in a way that induces unstructured precipitation.

It has been found recently that EGCG did not inhibit fibril formation according to ThT and TEM using the patient-derived λ-V_L_ sequence Mcg^29^. We would like to mention that similar controversies were also reported for Aβ and EGCG^56^. We speculate that this is related to a potentially lower amyloidogenicity of Mcg. Our results showed stronger effects of EGCG on more amyloidogenic mutants. For 6aJL2 R24G, the effect of EGCG on the ThT kinetics was reported already at lower stochiometries than observed here. This matches our expectations, as 6aJL2 R24G is known to form amyloid fibrils quite fast and thus might be more sensitive to EGCG.

**Figure 6:**
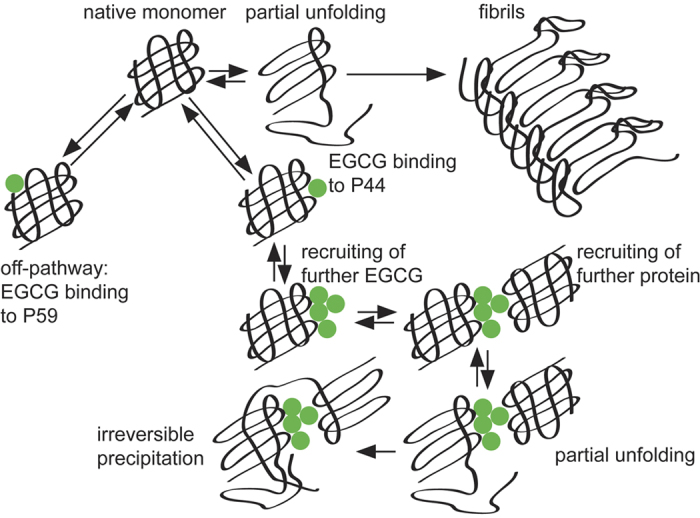
Mechanism of EGCG-induced V_L_ precipitation. Native monomers can be converted via partially unfolded intermediates into amyloid fibrils. EGCG can bind either to P59 (low affinity, no precipitation) or to P44 (higher affinity). More EGCG molecules are recruited to the P44 binding site, which in turn results in association of further proteins. Locally increased protein concentration and partial unfolding, as it presumably occurs prior to amyloid formation, are favourable conditions for precipitation.

Mutants with a higher amyloidogenicity induce faster precipitation. We were therefore wondering, whether it is possible to derive general principles to explain this difference. Hence, we suggest the following mechanism (Fig. 6). We assume that EGCG binds initially to a native V_L_ domain. EGCG can interact either with P44 or P59. The mutagenesis experiments suggest that interaction with the P59 site is not important for precipitation. By contrast, a more stably bound EGCG at P44 could recruit further EGCG molecules, considering its intrinsic tendency to form oligomers^57^. EGCG is mostly monomeric at the concentrations used here. However, also under these conditions, 1–2% of EGCG is in a dimeric state^58^. Similar models have been suggested earlier for binding of EGCG to β-casein^59^. Subsequently, two or more V_L_ domains, which are already bound to EGCG, could form a complex, with the self-associating EGCG molecules acting as a molecular glue. This complex should be still soluble, as both the native V_L_ domain and EGCG are rather hydrophilic. To explain the observed precipitation of the complex, V_L_ proteins must expose hydrophobic patches upon binding to EGCG. We suggest, this could be triggered by partial unfolding of already recruited V_L_ domains. Our results indicate that EGCG reacts stronger with more amyloidogenic mutants, even if they show similar thermodynamic stability as the wild-type. This could be explained by lowly populated partially folded intermediates on pathway to fibril formation, which are more often sampled by more amyloidogenic species^60^,^61^,^62^. A recent study on β2-microglobulin has investigated in detail such point mutants, which have reduced kinetic barriers for fibril formation, while at the same time the thermodynamic stability of the native state was not affected^63^. In AL amyloidosis, the C-C’ loop around P44 is presumably involved in initial steps of amyloid formation^22^,^24^. In such an early intermediate, the P44 binding site might be recognised by EGCG. Similarly, a higher affinity of EGCG for unfolded states has been reported earlier for BSA^41^. If several V_L_ proteins are already recruited to a cluster of EGCG molecules, the likelihood that multiple monomers enter such an intermediate state at the same time increases. Exposure of hydrophobic patches then could initiate aggregation and precipitation. The final result is a particle with EGCG in its center and a number of aggregated proteins covering it.

Drug discovery in AL amyloidosis is hampered by specificity. Potential drug candidates should prevent amyloid formation, but are not supposed to interfere with the necessary activity of antibodies. EGCG, despite being infamous as a promiscuous ligand, fulfills these requirements: It precipitates preferentially the amyloidogenic V_L_ domains. A similar selectivity of EGCG for amyloidogenic mutants was recently reported for TGFBIp^64^. At the same time, the EGCG binding site in functional antibodies is hidden, as the binding site is protected by the heavy chain. In LC dimers, which are known to be incompatible with amyloid formation^65^,^66^, the EGCG binding site is also not accessible.

We consider EGCG a valuable lead structure for further drug development, also considering the plethora of literature reports for its activity against different amyloidogenic proteins. Much progress has been made recently with issues concerning the *in vivo* stability of EGCG^67^,^68^,^69^: Different crystal forms or nanoparticles as transporters help to increase bioavailability. Even without special formulations, a one time oral application of 500 mg EGCG yields a plasma concentration of ca. 2 μM of free EGCG^70^. The median value for serum concentration of free λ-LCs in a cohort of AL-patients was ca. 160 mg/l^71^. Assuming an average V_L_ MW of 12 kDa, this corresponds to 13 μM, hence EGCG could already be bio-available at comparable concentrations. The question of *in vivo* activity is currently adressed by the clinical trials EpiCardiAL and TAME-AL (EpiCardiAL, ClinicalTrials.gov Identifier: NCT01511263; TAME-AL, ClinicalTrials.gov Identifier: NCT02015312) and it remains to be seen, how effective EGCG is as a therapeutic agent.

## Material and Methods

If not specified otherwise, all chemicals were purchased from Sigma Aldrich (Taufkirchen, Germany). EGCG (Sunphenon EGCG) was a kind gift by Stefan Schönland, supplied by Taiyo (Yokkaichi, Japan). All EGCG solutions were prepared freshly from EGCG powder. Aqueous stock solutions of EGCG were set to 20 mM and sonified to ensure solubilisation.

### Protein expression and purification

MAK33 V_L_ WT was expressed and purified as described previously^25^. Briefly, *E. coli* BL21 cells were grown in M9 minimal medium to OD_600_ = 0.6–0.8. Expression was then induced with 1 mM IPTG, using a pET28b vector. After expression over night at 37 °C, cells were harvested and inclusion bodies were separated from the soluble fraction. The inclusion bodies were dissolved in 25 mM Tris, 5 mM EDTA, 8 M urea and 2 mM β-mercaptoethanol and purified as the flow-through fraction by ion exchange chromatography with a 20 ml Q Sepharose column (GE Healthcare, Munich, Germany). Refolding was achieved by dialysis over night into 250 mM Tris, 100 mM L-arginine, 1 mM oxidized glutathione and 0.5 mM reduced glutathione (pH 8.0, 4 °C). The protein was purified by gel filtration using a 120 ml Superdex 75 column (GE Healthcare, Munich, Germany). As elution buffer, a solution of 20 mM sodium phosphate, 50 mM NaCl at pH 6.5 has been employed. For MAK33 V_L_ WT, the final yield after purification from 1 L M9 was on the order of 15–20 mg.

### Site-directed mutagenesis

Primers were purchased from Sigma-Aldrich (Munich, Germany). Mutagenesis protocols were adapted from the QuikChange site-directed mutagenesis kit (Agilent, Santa Clara, USA) using Pfu Ultra DNA polymerase (Agilent, Santa Clara, USA). After PCR, plasmids were digested with DpnI and then transformed into *E. coli* XL1.

### Solution-state NMR spectroscopy

Backbone assignments for MAK33 V_L_ WT have been reported previously^25^. Heteronuclear single quantum coherence (HSQC) experiments were recorded using a protein concentration of 50 μM at a temperature of 25 °C. All NMR experiments were performed using a Bruker Avance III spectrometer (Bruker BioSpin, Karlsruhe) operating at a ^1^H Larmor frequency of 500 MHz. The spectrometer was equipped with a triple resonance cryogenic probe. Experiments were recorded and processed using Topspin 3.2 (Bruker BioSpin, Karlsruhe) and analysed with CcpNMR Analysis 2^72^. Chemical shift perturbations were determined using the equation


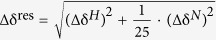


with δ being the chemical shift in ppm. PDB structures were analysed using UCSF Chimera 1.9^73^. EGCG-induced precipitation was quantified by comparing ^1^H 1D NMR signal intensities of V_L_ protein and EGCG. The indicated values are averages of triplicates, using the standard deviation as error. Samples were incubated in a thermoblock at 25 °C and 250 rpm between the short NMR experiments for measuring precipitation kinetics.

### Solid-state NMR spectroscopy

20-fold molar excess of EGCG was added to a 50 μM protein solution. This mixture was incubated for seven days, shaking at room temperature. Precipitates were sedimented at 21,000 g. An OptimaL-100 XP ultracentrifuge (Beckman Coulter, Krefeld, Germany) equipped with an SW 32 Ti swinging bucket rotor and a rotor filling device (Giotto Biotech, Florence, Italy) were used to pack the protein aggregate into an MAS rotor. The rotation frequency of the centrifuge was set to 28,000 rpm. Samples were packed into 3.2 mm thin wall ZrO_2_ rotors with vespel caps (CortecNet, Voisins Le Bretonneux, France) employing house-made teflon spacers.

Cross-polarization (CP) and proton driven spin diffusion (PDSD) experiments were recorded using a 500 MHz Bruker Avance II spectrometer (Bruker BioSpin, Karlsruhe) or a 750 MHz Bruker Avance III spectrometer (Bruker BioSpin, Karlsruhe). Both spectrometers are equipped with triple-resonance MAS probes. The magic angle spinning frequency was set to 11 kHz and 10 kHz, respectively. The set temperature of the probe was adjusted to 0 °C. For ^13^C,^13^C mixing, a PDSD mixing time of 50 ms was employed.

### Melting curves

Melting temperatures were determined using circular dichroism (CD) spectroscopy. Measurements were performed using a J715 spectropolarimeter (Jasco, Gross-Umstadt, Germany). Melting curves were recorded at a wavelength of 212 nm at a scan rate of 20 °C/h at 10 μM protein concentration. Curves were fitted with Matlab version R2014b (TheMathWorks, Natick, USA) using the equation


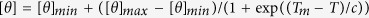


with *[θ]* being the ellipticity, *T*_*m*_ being the melting temperature and *c* being the cooperativity index.

### Transmission electron microscopy

Copper grids with 300 meshes coated with formvar/carbon film (Electron Microscopy Sciences, Hatfield, USA) were glow-discharged in argon atmosphere for 30 s at 3 mA. 5 μl of a 50 μM protein sample were incubated for 60 s on the grid. After removing the protein solution, the grid was washed with water. 5 μl uranyl acetate solution (2% w/v) were applied on the grid for staining and removed after 30 s. Photographs were taken on a Jeol JEM 100CX transmission electron microscope (Jeol, Tokio, Japan) operating at 100 kV. Images were recorded on Kodak SO163 films. Photographs were scanned at a resolution of 1000 dpi.

### Docking and MD simulations

Docking simulations of EGCG binding to MAK33 V_L_ were performed using both AutoDock Vina 1.1.2^74^ and HADDOCK 2.1^75^. The simulations were started from the V_L_ structure of PDB code 1FH5^49^ (residues 2–109 of the LC, which from now are called 1–108) with the mutations E17D, S20N and Y87F. With both docking programs semi-flexible docking was carried out, allowing EGCG to be flexible while the protein is considered rigid. The docking simulation with AutoDock Vina 1.1.2 was performed with an exhaustiveness factor of 100 and the entire surface of the protein as a possible target for EGCG binding. The docking simulation with HADDOCK 2.1 was performed with I58 and P59 as predefined contacts for EGCG binding, as predicted from our NMR experiments. We therefore call the simulation based on docking with AutoDock Vina as unguided simulation, while the simulation with HADDOCK is referred to as NMR-guided simulation. Further docking simulations employing different parameters and constraints were performed with both programs but the resulting MAK33 V_L_-EGCG complexes turned out to be unstable in the subsequent molecular dynamics (MD) simulations and thus are not presented here.

To test the stability of the obtained complexes and for allowing MAK33 V_L_ to adjust to the EGCG bound to it, MD simulations were performed using Gromacs 4.6.4^76^. The systems were modeled with the Amber99sb-ildn force field^77^ and the Tip4p-Ew water model^78^. EGCG was parametrised using acpype^79^, with bonded and Lennard-Jones parameters taken from the general Amber force field (GAFF)^80^ and RESP charges calculated with antechamber^81^ from a HF/6–31 G* single point calculation after the geometry was optimised at the B3LYP/6–31 G* level. Before starting the MD simulations, we performed steepest descent energy minimisations. The systems were then equilibrated with a 1 ns NVT simulation followed by a 1 ns NPT simulation. Afterwards we performed 500 ns production runs. In all MD simulations a cutoff of 1.0 nm was used for short-range van der Waals and electrostatic interactions, while long-range electrostatic interactions were calculated using the particle mesh Ewald method^82^. Periodic boundary conditions and a 2 fs timestep were used. The temperature was kept constant at 310 K with the Nosé-Hoover thermostat^83^ and the pressure was kept constant at 1.0 bar using the Parrinello-Rahman barostat^84^. For the analysis, we discarded the first 20 ns of each simulation, considering it as equilibration. The trajectories were analysed using Gromacs tools^76^ and MDAnalysis 0.8.1^85^. Contacts between EGCG and the protein were considered when the distance between any atom of the two molecules was less than 0.3 nm.

### Thioflavin T assay

Thioflavin T (ThT) assays were performed in triplicates in 96 medium binding well microplates (Greiner Bio-One, Frickenhausen, Germany) with a FP-8500 fluorescence spectrometer (Jasco, Gross-Umstadt, Germany) equipped with an FMP-825 fluorescence microplate reader (Jasco, Gross-Umstadt, Germany). Assays were conducted with 50 μM MAK33 V_L_ I2E protein solution, 10 μM ThT solution and EGCG from 0 to 250 μM (0 to 5 equivalents) in PBS buffer pH 7.4 with 0.05% NaN_3_ with a final reaction volume of 250 μl. ThT fluorescence was recorded at 440/482 nm excitation/emission wavelengths. Between the fluorescence measurements, 96 well microplates were incubated at 37 °C under continuous orbital shaking (350 rpm) using a PHMP thermoshaker (Grant Instruments, Cambridge, UK).

### Sequence analysis

Protein sequence alignments were calculated in Jalview 2.8.2^86^ using Clustal W^87^. The likelihood for a particular amino acid to occur at a specific positions was taken from the database abYsis 2.3.3. (http://www.bioinf.org.uk/abysis/). Amino acid frequencies were calculated for human LC sequences, where the number of available sequences varied between 11,000 and 15,000, depending on residue. Amino acid frequency plots were created with WebLogo 2.8.2, using the frequency plot option^88^.

## Additional Information

**How to cite this article**: Hora, M. *et al*. Epigallocatechin-3-gallate preferentially induces aggregation of amyloidogenic immunoglobulin light chains. *Sci. Rep.* **7**, 41515; doi: 10.1038/srep41515 (2017).

**Publisher's note:** Springer Nature remains neutral with regard to jurisdictional claims in published maps and institutional affiliations.

## Supplementary Material

Supporting Information

## References

[b1] Ramirez-Alvarado M. Amyloid Formation in Light Chain Amyloidosis. Curr. Top. Med. Chem. 12, 2523–2533 (2012). 23339305 10.2174/1568026611212220007 PMC3606678

[b2] Terry W. D. . Structural Identity of Bence Jones and Amyloid Fibril Proteins in a Patient with Plasma Cell Dyscrasia and Amyloidosis. J. Clin. Invest. 52, 1276–1281 (1973). 4700495 10.1172/JCI107295 PMC302384

[b3] Gertz M. a. . Definition of organ involvement and treatment response in immunoglobulin light chain amyloidosis (AL): a consensus opinion from the 10th International Symposium on Amyloid and Amyloidosis, Tours, France, 18–22 April 2004. Am. J. Hematol. 79, 319–328 (2005). 16044444 10.1002/ajh.20381

[b4] Desport E. . AL Amyloidosis. Orphanet J. Rare Dis. 7, 54 (2012). 22909024 10.1186/1750-1172-7-54 PMC3495844

[b5] Merlini G., Seldin D. C. & Gertz M. a. Amyloidosis: Pathogenesis and New Therapeutic Options. J. Clin. Oncol. 29, 1924–1933 (2011). 21483018 10.1200/JCO.2010.32.2271 PMC3138545

[b6] Kumar S. K. . Recent Improvements in Survival in Primary Systemic Amyloidosis and the Importance of an Early Mortality Risk Score. Mayo Clin. proceedings. 86, 12–18 (2011). 10.4065/mcp.2010.0480 PMC3012628 21193650

[b7] Girnius S. . Safety and efficacy of high-dose melphalan and auto-SCT in patients with AL amyloidosis and cardiac involvement. Bone Marrow Transplant. 49 (2014). 10.1038/bmt.2013.192 24317129

[b8] Schönland S. O., Dreger P., de Witte T. & Hegenbart U. Current status of hematopoietic cell transplantation in the treatment of systemic amyloid light-chain amyloidosis. Bone Marrow Transplant. 47, 895–905 (2012). 21785469 10.1038/bmt.2011.152

[b9] Reece D. E. . Long-term follow-up from a phase 1/2 study of single-agent bortezomib in relapsed systemic AL amyloidosis. Blood 124, 2498–2506 (2014). 25202139 10.1182/blood-2014-04-568329 PMC4199951

[b10] Cornell R. F. . Bortezomib-based induction for transplant ineligible AL amyloidosis and feasibility of later transplantation. Bone Marrow Transplant. 50, 914–917 (2015). 25915809 10.1038/bmt.2015.73

[b11] Reece D. E. . Weekly and twice-weekly bortezomib in patients with systemic AL amyloidosis: results of a phase 1 dose-escalation study. Blood 114, 1489–1497 (2010). 10.1182/blood-2009-02-203398 19498019

[b12] Olsen K. E., Sletten K. & Westermark P. Fragments of the constant region of immunoglobulin light chains are constituents of AL-amyloid proteins. Biochem. Biophys. Res. Commun. 251, 642–647 (1998). 9792827 10.1006/bbrc.1998.9508

[b13] Klimtchuk E. S. . The critical role of the constant region in thermal stability and aggregation of amyloidogenic immunoglobulin light chain. Biochemistry 49, 9848–9857 (2010). 20936823 10.1021/bi101351c PMC4080313

[b14] Nokwe C. N. . The Antibody Light-Chain Linker Is Important for Domain Stability and Amyloid Formation. J. Mol. Biol. 427, 3572–3586 (2015). 26408269 10.1016/j.jmb.2015.09.012

[b15] Comenzo R. L., Zhang Y., Martinez C., Osman K. & Herrera G. a. The tropism of organ involvement in primary systemic amyloidosis: Contributions of Ig V L germ line gene use and clonal plasma cell burden. Blood 98, 714–720 (2001). 11468171 10.1182/blood.v98.3.714

[b16] Abraham R. S. . Immunoglobulin light chain variable (V) region genes influence clinical presentation and outcome in light chain-associated amyloidosis (AL). Blood 101, 3801–3807 (2003). 12515719 10.1182/blood-2002-09-2707

[b17] Poshusta T. L., Katoh N., Gertz M. a., Dispenzieri A. & Ramirez-Alvarado M. Thermal stability threshold for amyloid formation in light chain amyloidosis. Int. J. Mol. Sci. 14, 22604–22617 (2013). 24248061 10.3390/ijms141122604 PMC3856080

[b18] Martsev S. P. . Amyloid fibril formation of the mouse V(L) domain at acidic pH. Biochemistry 41, 3389–3395 (2002). 11876647 10.1021/bi015894u

[b19] Simpson E. R., Herold E. M. & Buchner J. The folding pathway of the antibody V(L) domain. J. Mol. Biol. 392, 1326–1338 (2009). 19647749 10.1016/j.jmb.2009.07.075

[b20] Feige M. J. . Dissecting the alternatively folded state of the antibody Fab fragment. J. Mol. Biol. 399, 719–730 (2010). 20434459 10.1016/j.jmb.2010.04.032

[b21] Mukherjee S., Pondaven S. P. & Jaroniec C. P. Chain Variable Domain by Relaxation Dispersion Nuclear Magnetic and Amyloid Assembly. Biochemistry 50, 5845–5857 (2011). 21627161 10.1021/bi200410c

[b22] Poshusta T. L. . Mutations in specific structural regions of immunoglobulin light chains are associated with free light chain levels in patients with AL amyloidosis. PLoS One 4, e5169 (2009). 19365555 10.1371/journal.pone.0005169 PMC2664898

[b23] Wall J. S. . Structural basis of light chain amyloidogenicity: comparison of the thermodynamic properties, fibrillogenic potential and tertiary structural features of four Vλ6 proteins. J. Mol. Recognit. 17, 323–331 (2004). 15227639 10.1002/jmr.681

[b24] Baden E. M., Randles E. G., Aboagye A. K., Thompson J. R. & Ramirez-Alvarado M. Structural insights into the role of mutations in amyloidogenesis. J. Biol. Chem. 283, 30950–30956 (2008). 18768467 10.1074/jbc.M804822200 PMC2576559

[b25] Nokwe C. N. . A Residue-specific Shift in Stability and Amyloidogenicity of Antibody Variable Domains. J. Biol. Chem. 289, 26829–26846 (2014). 25096580 10.1074/jbc.M114.582247 PMC4175325

[b26] Nokwe C. N. . A Stable Mutant Predisposes Antibody Domains to Amyloid Formation through Specific Non-Native Interactions. J. Mol. Biol. 428, 1315–1332 (2016). 26827727 10.1016/j.jmb.2016.01.015

[b27] Villalba M. I. . Site-directed Mutagenesis Reveals Regions Implicated in the Stability and Fiber Formation of Human λ3r Light Chains. J. Biol. Chem. 290, 2577–2592 (2014). 25505244 10.1074/jbc.M114.629550 PMC4317031

[b28] Richards D. B. . Therapeutic Clearance of Amyloid by Antibodies to Serum Amyloid P Component. N. Engl. J. Med. 373, 1106–1114 (2015). 26176329 10.1056/NEJMoa1504942

[b29] Brumshtein B. . Inhibition by small-molecule ligands of formation of amyloid fibrils of an immunoglobulin light chain variable domain. Elife 4, e10935 (2015). 26576950 10.7554/eLife.10935 PMC4758944

[b30] Lorenzen N. . How epigallocatechin gallate can inhibit α-synuclein oligomer toxicity *in vitro*. J. Biol. Chem. 289, 21299–21310 (2014). 24907278 10.1074/jbc.M114.554667 PMC4118093

[b31] Lopez del Amo J. M. . Structural properties of EGCG-induced, nontoxic Alzheimer’s disease Aβ oligomers. J. Mol. Biol. 421, 517–524 (2012). 22300765 10.1016/j.jmb.2012.01.013

[b32] Hyung S.-J. . Insights into antiamyloidogenic properties of the green tea extract (-)-epigallocatechin-3-gallate toward metal-associated amyloid- species. Proc. Natl. Acad. Sci. 110, 3743–3748 (2013). 23426629 10.1073/pnas.1220326110 PMC3593904

[b33] Ehrnhoefer D. E. . Green tea (-)-epigallocatechin-gallate modulates early events in huntingtin misfolding and reduces toxicity in Huntington’s disease models. Hum. Mol. Genet. 15, 2743–2751 (2006). 16893904 10.1093/hmg/ddl210

[b34] Suzuki Y., Brender J. R., Hartman K., Ramamoorthy A. & Marsh E. N. G. Alternative pathways of human islet amyloid polypeptide aggregation distinguished by 19F nuclear magnetic resonance-detected kinetics of monomer consumption. Biochemistry 51, 8154–8162 (2012). 22998665 10.1021/bi3012548 PMC3543753

[b35] Wang Q., Guo J., Jiao P., Liu H. & Yao X. Exploring the Influence of EGCG on the β-Sheet-Rich Oligomers of Human Islet Amyloid Polypeptide (hIAPP1-37) and Identifying Its Possible Binding Sites from Molecular Dynamics Simulation. PLoS One 9, e94796 (2014). 24739876 10.1371/journal.pone.0094796 PMC3989243

[b36] Miyata M. . The crystal structure of the green tea polyphenol (-)-epigallocatechin gallate-transthyretin complex reveals a novel binding site distinct from the thyroxine binding site. Biochemistry 49, 6104–6114 (2010). 20565072 10.1021/bi1004409

[b37] Trivella D. B. B., dos Reis C. V., Lima L. M. T. R., Foguel D. & Polikarpov I. Flavonoid interactions with human transthyretin: combined structural and thermodynamic analysis. J. Struct. Biol. 180, 143–153 (2012). 22842046 10.1016/j.jsb.2012.07.008

[b38] Wobst H. J., Sharma A., Diamond M. I., Wanker E. E. & Bieschke J. The green tea polyphenol (-)-epigallocatechin gallate prevents the aggregation of tau protein into toxic oligomers at substoichiometric ratios. FEBS Lett. 589, 77–83 (2014). 25436420 10.1016/j.febslet.2014.11.026 PMC4565179

[b39] Popovych N. . Site specific interaction of the polyphenol EGCG with the SEVI amyloid precursor peptide PAP (248–286). J. Phys. Chem. B 116, 3650–3658 (2012). 22360607 10.1021/jp2121577 PMC3310975

[b40] Palhano F. L., Lee J., Grimster N. P. & Kelly J. W. Toward the Molecular Mechanism(s) by which EGCG Treatment Remodels Mature Amyloid Fibrils. J. Am. Chem. Soc. 135, 7503–7510 (2013). 23611538 10.1021/ja3115696 PMC3674815

[b41] Ehrnhoefer D. E. . EGCG redirects amyloidogenic polypeptides into unstructured, off-pathway oligomers. Nat. Struct. Mol. Biol. 15, 558–566 (2008). 18511942 10.1038/nsmb.1437

[b42] Bieschke J. . EGCG remodels mature alpha-synuclein and amyloid-beta fibrils and reduces cellular toxicity. Proc. Natl. Acad. Sci. USA 107, 7710–7715 (2010). 20385841 10.1073/pnas.0910723107 PMC2867908

[b43] Andrich K. & Bieschke J. Natural Compounds as Therapeutic Agents for Amyloidogenic Diseases. Adv. Exp. Med. Biol. 863, 139–161 (2015). 26092630 10.1007/978-3-319-18365-7_7 PMC5891833

[b44] Pelaez-Aguilar A. . Inhibition of light chain 6aJL2-R24G amyloid fiber formation associated with AL amyloidosis. Biochemistry 54, 4978–4986 (2015). 26214579 10.1021/acs.biochem.5b00288

[b45] Diomede L. . A Caenorhabditis elegans-based assay recognizes immunoglobulin light chains causing heart amyloidosis. Blood 123, 3543–3552 (2014). 24665135 10.1182/blood-2013-10-525634 PMC4047494

[b46] Hunstein W. Epigallocathechin-3-gallate in AL amyloidosis: a new therapeutic option? Blood 110, 2216 (2007). 17785589 10.1182/blood-2007-05-089243

[b47] Mereles D., Wanker E. E. & Katus H. a. Therapy effects of green tea in a patient with systemic light-chain amyloidosis. Clin. Res. Cardiol. 97, 341–344 (2008). 18317666 10.1007/s00392-008-0649-6

[b48] Mereles D., Buss S. J., Hardt S. E., Hunstein W. & Katus H. a. Effects of the main green tea polyphenol epigallocatechin-3-gallate on cardiac involvement in patients with AL amyloidosis. Clin. Res. Cardiol. 99, 483–490 (2010). 20221615 10.1007/s00392-010-0142-x

[b49] Augustine J. G., de La Calle A., Knarr G., Buchner J. & Frederick C. a. The crystal structure of the Fab fragment of the monoclonal antibody MAK33. Implications for folding and interaction with the chaperone bip. J. Biol. Chem. 276, 3287–3294 (2001). 11036070 10.1074/jbc.M005221200

[b50] Charlton A. J. . Polyphenol/Peptide Binding and Precipitation. J. Agric. Food Chem. 50, 1593–1601 (2002). 11879042 10.1021/jf010897z

[b51] Hagerman A. E. & Butler L. G. The specificity of proanthocyanidin-protein interactions. J. Biol. Chem. 256, 4494–4497 (1981). 7217094

[b52] Kaplan B., Livneh A. & Sela B.-A. Immunoglobulin free light chain dimers in human diseases. ScientificWorldJournal. 11, 726–735 (2011). 21442150 10.1100/tsw.2011.65 PMC5720091

[b53] Tuttle M. D., Courtney J. M., Barclay A. M. & Rienstra C. M. Preparation of Amyloid Fibrils for Magic-Angle Spinning Solid-State NMR Spectroscopy. Methods Mol. Biol. 1345, 173–183 (2016). 26453212 10.1007/978-1-4939-2978-8_11

[b54] Schubert M., Labudde D., Oschkinat H. & Schmieder P. A software tool for the prediction of Xaa-Pro peptide bond conformations in proteins based on 13C chemical shift statistics. J. Biomol. NMR 24, 149–154 (2002). 12495031 10.1023/a:1020997118364

[b55] Groenning M. . Binding mode of Thioflavin T in insulin amyloid fibrils. J. Struct. Biol. 159, 483–497 (2007). 17681791 10.1016/j.jsb.2007.06.004

[b56] Šneideris T. . Looking for a generic inhibitor of amyloid-like fibril formation among flavone derivatives. PeerJ 3, e1271 (2015). 26421240 10.7717/peerj.1271 PMC4586895

[b57] Wroblewski K., Muhandiram R., Chakrabartty A. & Bennick A. The molecular interaction of human salivary histatins with polyphenolic compounds. Eur. J. Biochem. 268, 4384–4397 (2001). 11502198 10.1046/j.1432-1327.2001.02350.x

[b58] Ujihara T. & Hayashi N. Association of Catechin Molecules in Water: Quantitative Binding Study and Complex Structure Analysis. J. Nat. Prod. 79, 66–73 (2015). 26720794 10.1021/acs.jnatprod.5b00658

[b59] Jöbstl E., O’Connell J. & Fairclough J. P. a & Williamson, M. P. Molecular model for astringency produced by polyphenol/protein interactions. Biomacromolecules 5, 942–949 (2004). 15132685 10.1021/bm0345110

[b60] Light I., Len C., Souillac P. O., Uversky V. N. & Fink A. L. Structural Transformations of Oligomeric Intermediates in the Fibrillation of the Immunoglobulin Light Chain LEN. Biochemistry 42, 8094–8104 (2003). 12834361 10.1021/bi034652m

[b61] Qin Z., Hu D., Zhu M. & Fink A. L. Structural characterization of the partially folded intermediates of an immunoglobulin light chain leading to amyloid fibrillation and amorphous aggregation. Biochemistry 46, 3521–3531 (2007). 17315948 10.1021/bi061716v

[b62] Blancas-Mejia L. M. . Thermodynamic and kinetic characterization of a germ line human lambda6 light-chain protein: the relation between unfolding and fibrillogenesis. J. Mol. Biol. 386, 1153–1166 (2009). 19154739 10.1016/j.jmb.2008.12.069

[b63] Camilloni C. . Rational design of mutations that change the aggregation rate of a protein while maintaining its native structure and stability. Sci. Rep. 6 (2016). 10.1038/srep25559 PMC4858664 27150430

[b64] Stenvang M., Christiansen G. & Otzen D. E. Epigallocatechin gallate remodels fibrils of Lattice Corneal Dystrophy protein, facilitating proteolytic degradation and preventing formation of membrane-permeabilizing species. Biochemistry 55, 2344–2357 (2016). 27042751 10.1021/acs.biochem.6b00063

[b65] Brumshtein B. . Formation of Amyloid Fibers by Monomeric Light-chain Variable Domains. J. Biol. Chem. 289, 27513–27525 (2014). 25138218 10.1074/jbc.M114.585638 PMC4183792

[b66] Wolwertz M. L., Nguyen P. T., Quittot N. & Bourgault S. Probing the role of λ6 immunoglobulin light chain dimerization in amyloid formation. Biochim. Biophys. Acta - Proteins Proteomics 1864, 409–418 (2016). 10.1016/j.bbapap.2016.01.009 26802902

[b67] Hu B., Ting Y., Zeng X. & Huang Q. Cellular uptake and cytotoxicity of chitosan-caseinophosphopeptides nanocomplexes loaded with epigallocatechin gallate. Carbohydr. Polym. 89, 362–370 (2012). 24750731 10.1016/j.carbpol.2012.03.015

[b68] Smith A. J. . Crystal engineering of green tea epigallocatechin-3-gallate (EGCg) cocrystals and pharmacokinetic modulation in rats. Mol. Pharm. 10, 2948–2961 (2013). 23730870 10.1021/mp4000794 PMC3795472

[b69] Zhang J. . Epigallocatechin-3-gallate (EGCG) stabilized selenium nanoparticles coated with Tet-1 peptide reduce amyloid-β aggregation and cytotoxicity. ACS Appl. Mater. Interfaces 6, 8475–8487 (2014). 24758520 10.1021/am501341u

[b70] Naumovski N., Blades B. & Roach P. Food Inhibits the Oral Bioavailability of the Major Green Tea Antioxidant Epigallocatechin Gallate in Humans. Antioxidants 4, 373–393 (2015). 26783711 10.3390/antiox4020373 PMC4665468

[b71] Nelson L. M., Gustafsson F. & Gimsing P. Characteristics and Long-Term Outcome of Patients with Systemic Immunoglobulin Light-Chain Amyloidosis. Acta Haematol. 133, 336–346 (2014). 25531398 10.1159/000363682

[b72] Vranken W. F. . The CCPN data model for NMR spectroscopy: Development of a software pipeline. Proteins Struct. Funct. Bioinforma. 59, 687–696 (2005). 10.1002/prot.20449 15815974

[b73] Pettersen E. F. . UCSF Chimera - A visualization system for exploratory research and analysis. J. Comput. Chem. 25, 1605–1612 (2004). 15264254 10.1002/jcc.20084

[b74] Trott O. & Olson A. AutoDock Vina: improving the speed and accuracy of docking with a new scoring function, efficient optimization and multithreading. J. Comput. Chem. 31, 455–461 (2010). 19499576 10.1002/jcc.21334 PMC3041641

[b75] de Vries S. J., van Dijk M. & Bonvin A. M. J. J. The HADDOCK web server for data-driven biomolecular docking. Nat. Protoc. 5, 883–897 (2010). 20431534 10.1038/nprot.2010.32

[b76] Hess B., Kutzner C., Van Der Spoel D. & Lindahl E. GROMACS 4: Algorithms for highly efficient, load-balanced, and scalable molecular simulation. J. Chem. Theory Comput. 4, 435–447 (2008). 26620784 10.1021/ct700301q

[b77] Lindorff-Larsen K. . Improved side-chain torsion potentials for the Amber ff99SB protein force field. Proteins Struct. Funct. Bioinforma. 78, 1950–1958 (2010). 10.1002/prot.22711 PMC2970904 20408171

[b78] Horn H. W. . Development of an improved four-site water model for biomolecular simulations: TIP4P-Ew. J. Chem. Phys. 120, 9665–9678 (2004). 15267980 10.1063/1.1683075

[b79] Sousa da Silva A. W. & Vranken W. F. ACPYPE - AnteChamber PYthon Parser interfacE. BMC Res. Notes 5, 367 (2012). 22824207 10.1186/1756-0500-5-367 PMC3461484

[b80] Wang J., Wang W., Kollman P. a & Case D. a. Development and testing of a general amber force field. J. Comput. Chem 25, 1157–1174 (2004). 15116359 10.1002/jcc.20035

[b81] Wang J., Wang W., Kollman P. a. & Case D. a. Automatic atom type and bond type perception in molecular mechanical calculations. J. Mol. Graph. Model. 25, 247–260 (2006). 16458552 10.1016/j.jmgm.2005.12.005

[b82] Darden T., York D. & Pedersen L. Particle mesh Ewald: An N⋅log(N) method for Ewald sums in large systems. J. Chem. Phys. 98, 10089–10092 (1993).

[b83] Nosé S. A unified formulation of the constant temperature molecular dynamics methods. J. Chem. Phys. 81, 511–519 (1984).

[b84] Parrinello M. & Rahman a. Polymorphic transitions in single crystals: A new molecular dynamics method. J. Appl. Phys. 52, 7182–7190 (1981).

[b85] Michaud-Agrawal N., Denning E. J., Woolf T. B. & Beckstein O. MDAnalysis: A toolkit for the analysis of molecular dynamics simulations. J. Comput. Chem. 32, 2319–2327 (2011). 21500218 10.1002/jcc.21787 PMC3144279

[b86] Waterhouse a. M., Procter J. B., Martin D. M. a., Clamp M. & Barton G. J. Jalview Version 2-a multiple sequence alignment editor and analysis workbench. Bioinformatics 25, 1189–1191 (2009). 19151095 10.1093/bioinformatics/btp033 PMC2672624

[b87] Larkin M. a. . Clustal W and Clustal X version 2.0. Bioinformatics 23, 2947–2948 (2007). 17846036 10.1093/bioinformatics/btm404

[b88] Crooks G., Hon G., Chandonia J. & Brenner S. WebLogo: a sequence logo generator. Genome Res 14, 1188–1190 (2004). 15173120 10.1101/gr.849004 PMC419797

