# Supplementary material for: Epigallocatechin-3-gallate preferentially induces aggregation of amyloidogenic immunoglobulin light chains
Source: Sci Rep. 2017 Jan 27;7:41515. doi: 10.1038/srep41515 (PMC5269747; doi:10.1038/srep41515)
Supplement: Supporting Information [file srep41515-s1.pdf]

Supporting Information for the manuscript

Epigallocatechin-3-gallate preferentially induces aggregation of amyloidogenic immunoglobulin light chains

Manuel Hora[1,2], Martin Carballo Pacheco[3], Benedikt Weber[1], Vanessa K. Morris[1], Antje Wittkopf[1], Johannes Buchner[1], Birgit Strodel[3], Bernd Reif[1,2]*

Affiliations

1: Center for Integrated Protein Science at Department Chemie, Technische Universität München Lichtenbergstrasse 4, 85747 Garching, Germany

2: Helmholtz-Zentrum München (HMGU), Ingolstädter Landstr. 1, 85764 Neuherberg, Germany

3: Institute of Complex Systems: Structural Biochemistry at Forschungszentrum Jülich, 52425 Jülich, Germany

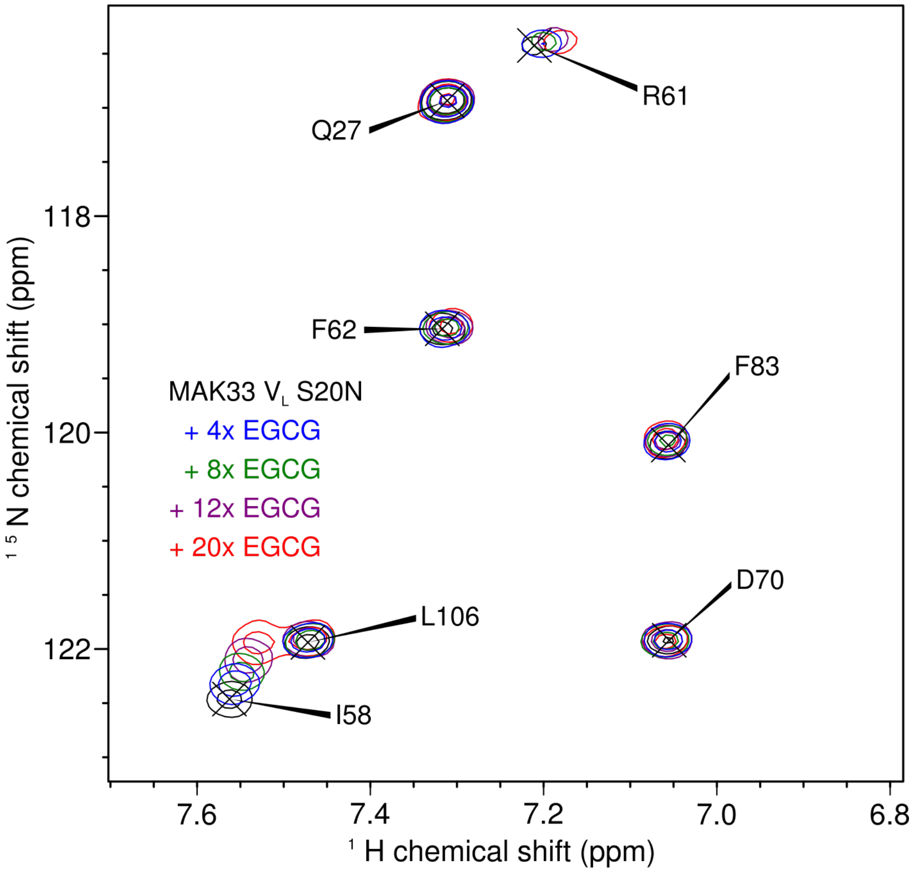

SI 1: Chemical shift changes at varying EGCG concentrations. EGCG was added stepwise to 50 µM MAK33 V_L S20N. A region of the ^{1}H,^{15}N NMR correlation spectra is shown.

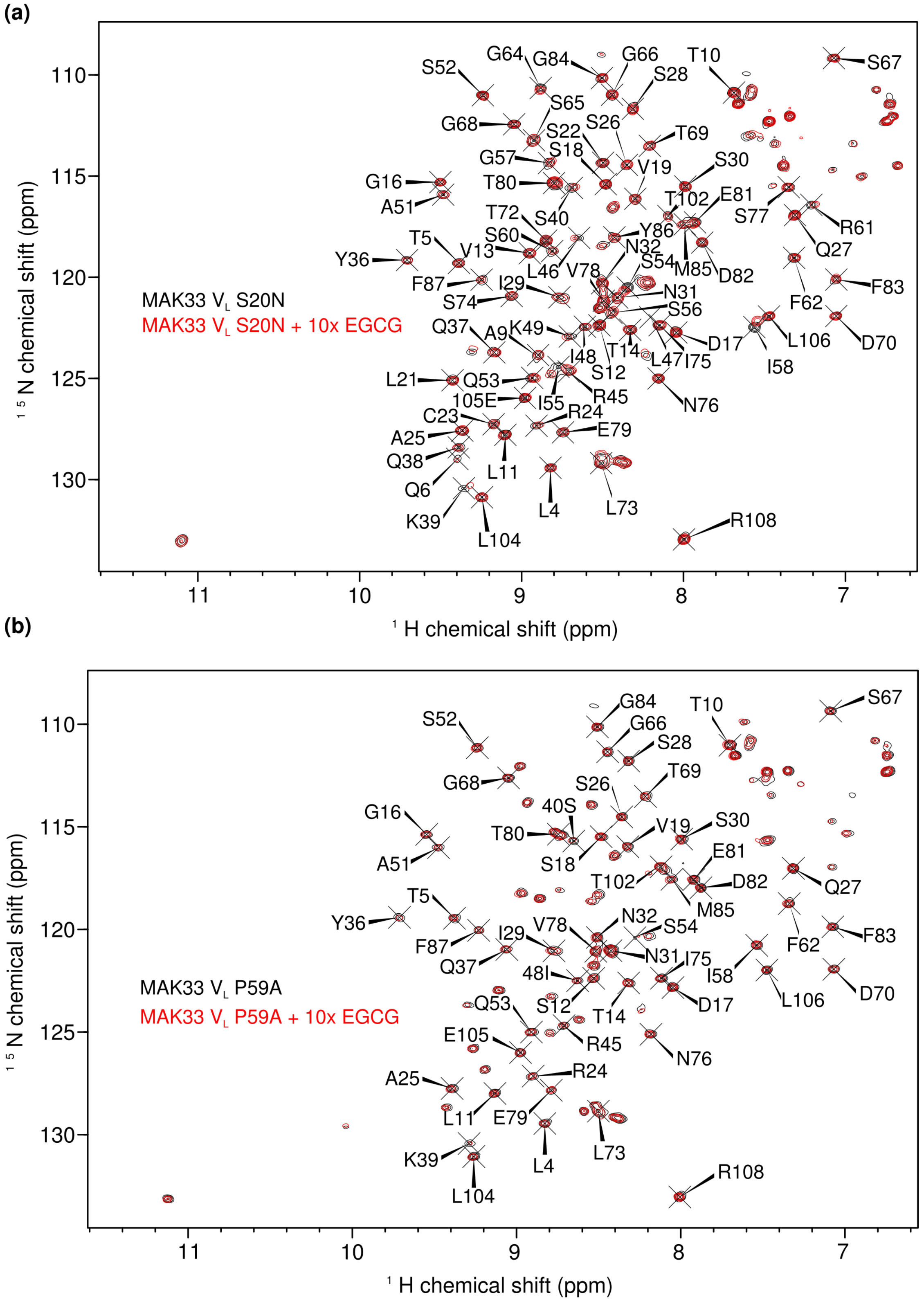

SI 2: $^{1}H,^{15}N$ NMR correlation spectra of MAK33 V_L S20N and V_L P59A in presence and absence of 10x molar excess of EGCG. The spectra shown here are the full HSQC experiments, of which a part is displayed in the main publication, Fig. 1b) and c).

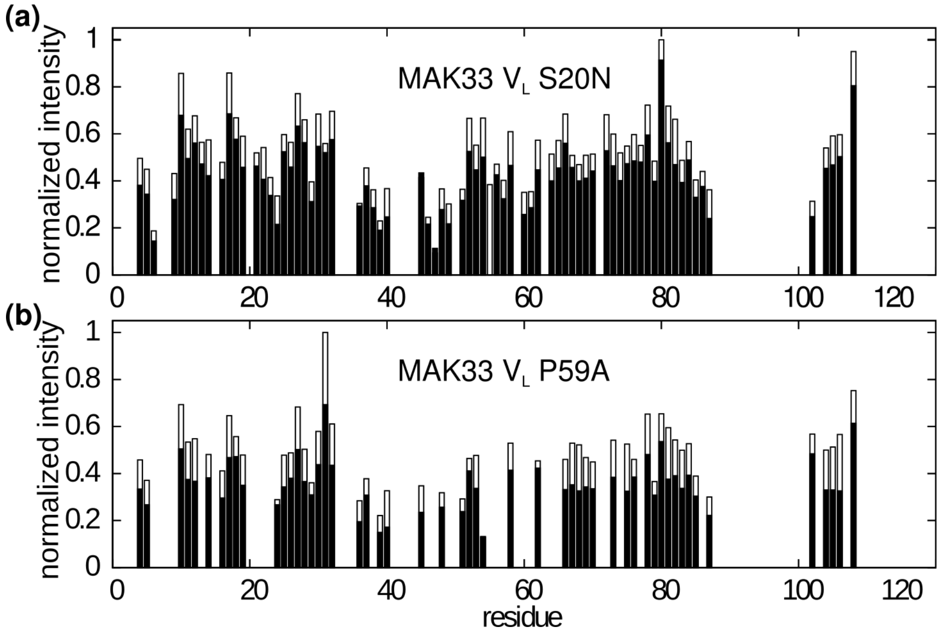

SI 3: Relative NMR signal intensities of MAK33 V_L S20N (a) and V_L P59A (b) in absence (white) and presence of 10x molar excess of EGCG (black).

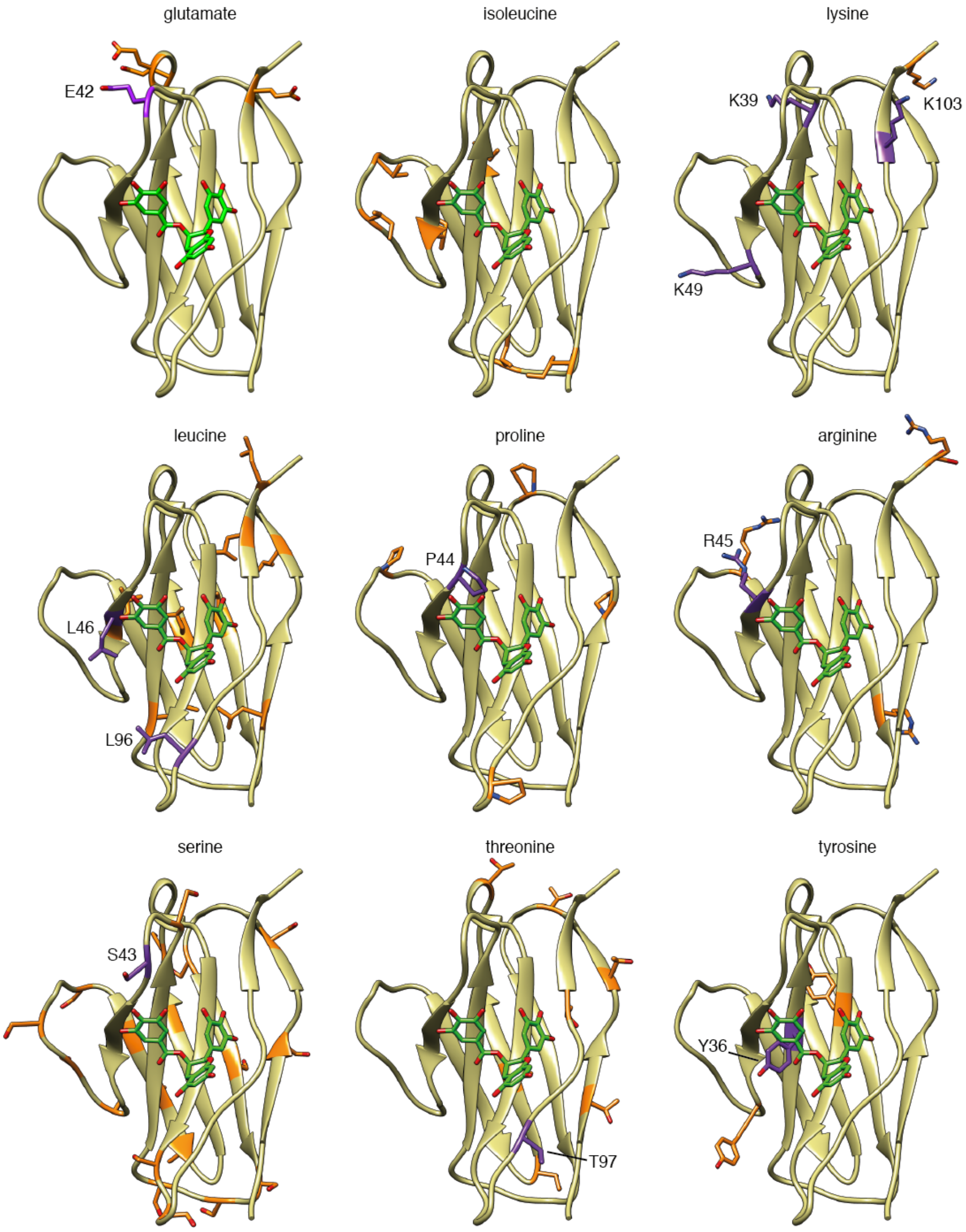


```
1          11         21         31         41         51
DIVLTQSPAT LSVTPGDSVS LSCRASQSIS NNLHWYQQKS HESPRLLIKY ASQSISGIPS
61         71         81         91         101
RFSGSGSGTD FTLSINSVET EDFGMYFCQQ SNSWPLTFGA GTKLELKR
```

SI 4: Residues identified by MAS solid-state NMR spectroscopy were assigned based on structural proximity to EGCG. The most stable binding pose of EGCG from the docking experiments was used for analysis. For each amino acid type, all residues are highlighted in orange. The closest residue to EGCG is highlighted in purple. In case of isoleucine, no close residue could be identified. For lysine und leucine, several conceivable residues were found. These ambiguous assignments are highlighted in yellow in the primary sequences. Unambiguous assignments are shown in green.

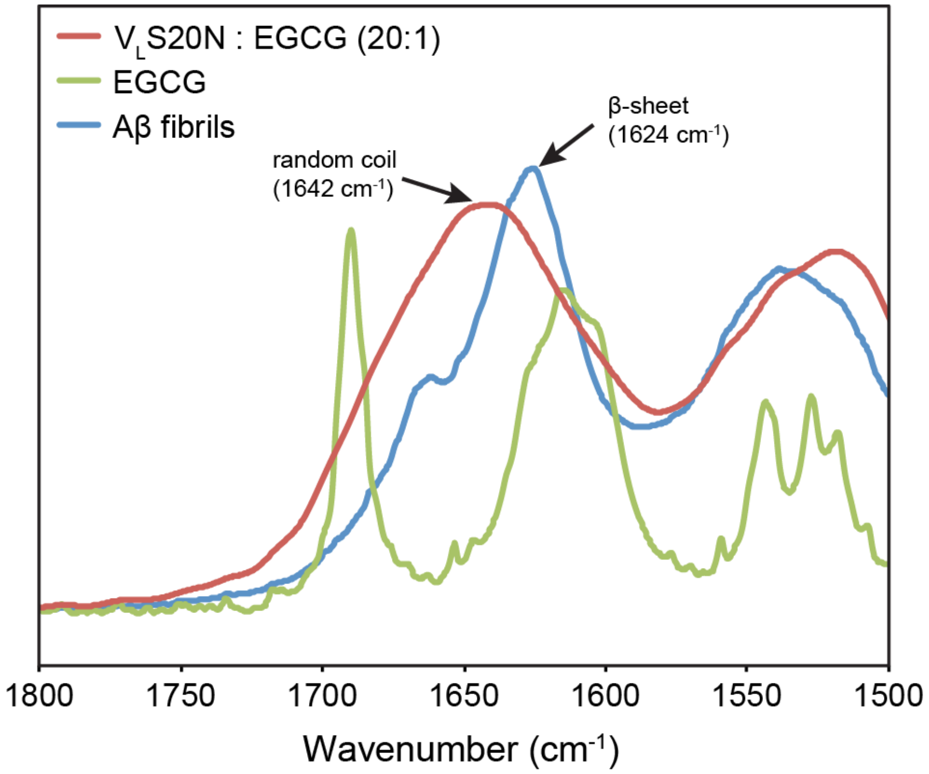

SI 5: Fourier-transform infrared spectroscopic (FTIR) analysis of EGCG-protein coprecipitates (20-fold molar excess of EGCG). Precipitates of MAK33 V_L S20N with EGCG (red) show an FTIR peak at 1642 cm^{-1}. Interpretation of the amide I peak is difficult due to overlap with EGCG signals (green). The peak at 1642 cm^{-1} indicates disordered or helical structure [1,2]. In comparison, amyloid-beta fibrils (blue) display a characteristic peak at 1624 cm^{-1}.

Material and methods

Fourier-transform infrared spectroscopy

EGCG was added to a solution of 50 µM MAK33 V_L S20N to a final concentration of 1 mM and incubated with shaking at room temperature for 4 days. The aggregates were isolated by centrifugation and washed once with water, and then lyophilised to remove residual water. Amyloid-beta fibrils were prepared from peptide recombinantly expressed and purified as previously reported [3]. Lyophilised peptide was dissolved in 10 mM NaOH and centrifuged to remove preformed aggregates, before dissolution in buffer to a final concentration of 50 µM amyloid-beta, 50 mM phosphate, pH 7.4. This solution was shaken at room temperature for one week. Fibrils were isolated by centrifugation and washed once with water. Spectra were recorded on a JASCO FT/IR-4100 FT-IR spectrometer with attenuated total reflectance (ATR) attachment. The samples were measured with 128 scans at a resolution of 2 cm^{-1} at room temperature.

References

1. Byler, D. M. & Susi, H. Examination of the secondary structure of proteins by deconvolved FTIR spectra. *Biopolymers* **25,** 469–487 (1986).

2. Fowler, D. M. *et al.* Functional amyloid formation within mammalian tissue. *PLoS Biol.* **4,** 0100–0107 (2006).

3. Dasari, M. *et al.* Bacterial inclusion bodies of Alzheimer’s disease β-amyloid peptides can be employed to study native-like aggregation intermediate states. *Chembiochem* **12,** 407–423 (2011).
